# Characterization of *Mycobacterium tuberculosis* isolates from Hebei, China: genotypes and drug susceptibility phenotypes

**DOI:** 10.1186/s12879-016-1441-2

**Published:** 2016-03-03

**Authors:** Yanan Li, Xinrui Cao, Shiming Li, Hao Wang, Jianlin Wei, Peng Liu, Jing Wang, Zhi Zhang, Huixia Gao, Machao Li, Kanglin Wan, Erhei Dai

**Affiliations:** Department of Laboratory Medicine, the Fifth Hospital of Shijiazhuang, Hebei Medical University, 42 Tanan Road, Shijiazhuang, Hebei 050021 China; Department of Laboratory Medicine, Cangzhou Infectious Disease Hospital, 68 Guangrong Road, Cangzhou, Hebei China; Department of Laboratory Medicine, the Fourth Hospital of Tangshan, South Xuejing Road, Tangshan, Hebei China; Department of Laboratory Medicine, Baoding Infectious Disease Hospital, 608 East Dongfeng Road, Baoding, Hebei China; Department of Laboratory Medicine, the Second Hospital of Xingtai, 371 Yurangqiao Road, Xingtai, Hebei China; Department of Laboratory Medicine, the Third Hospital of Qinhuangdao, 222 Jianguo Road, Qinhuangdao, Hebei China; Department of Laboratory Medicine, Affiliated Hospital of Hebei University, 212 East Yuhua Road, Baoding, Hebei China; State Key Laboratory for Infectious Disease Prevention and Control, National Institute for Communicable Disease Control and Prevention, Chinese Center for Disease Control and Prevention, 155 Changbai Road, Changping, Beijing, 102206 China

**Keywords:** *Mycobacterium tuberculosis*, MIRU-VNTR, Spoligotyping, Drug resistance

## Abstract

**Background:**

Tuberculosis remains a major public health problem in China. The Hebei province is located in the Beijing-Tianjin-Hebei integration region; however little information about the genetic diversity of *Mycobacterium tuberculosis* was available in this area. This study describes the first attempt to map the molecular epidemiology of MTB strains isolated from Hebei.

**Methods:**

Spoligotyping and 15-locus MIRU-VNTR were performed in combination to yield specific genetic profiles of 1017 MTB strains isolated from ten cities in the Hebei province in China during 2014. Susceptibility testing to first line anti-TB drugs was also conducted for all strains using the L-J proportion method.

**Results:**

Based on the SpolDB4.0 database, the predominant spoligotype belonged to the Beijing family (90.5 %), followed by T family (6.3 %). Using 15-locus MIRU-VNTR clustering analysis, 846 different patterns were identified, including 84 clusters (2–17 strains per cluster) and 764 individual types. Drug susceptibility pattern showed that 347 strains (34.1 %) were resistant to at least one of the first line drugs, including 134 (13.2 %) multi-drug resistance strains. Statistical analysis indicated that drug resistance was associated with treatment history. The Beijing family was associated with genetic clustering. However, no significant difference was observed between the Beijing and non-Beijing family in gender, age, treatment history and drug resistance.

**Conclusions:**

The *Mycobacterium tuberculosis* strains in Hebei exhibit high genetic diversity. The Beijing family is the most prevalent lineage in this area. Spoligotyping in combination with 15-locus MIRU-VNTR is a useful tool to study the molecular epidemiology of the MTB strains in Hebei.

**Electronic supplementary material:**

The online version of this article (doi:10.1186/s12879-016-1441-2) contains supplementary material, which is available to authorized users.

## Background

Tuberculosis (TB) remains a major public health challenge facing the world, second only to HIV/AIDS as the greatest killer worldwide due to a single infectious agent. According to a report from the World Health Organization (WHO), approximately one-third of the world’s population has latent TB [1, 2]. In 2013, 9 million people fell ill with TB and 1.5 million died from this disease globally, with over 95 % of the TB deaths occurring in low- and middle-income countries. China, the largest developing country, has the second highest TB burden after India with 1.3 million TB patients and more than 0.8 million new cases in 2013. Approximately 5.7 % of new infections and 26 % of retreated patients developed into multi-drug resistance TB (MDR-TB) cases [2]. Despite the decrease in morbidity and mortality rates, China still has a long way to go to control TB due to the huge infected population base and the lack of medical facilities and other social restrictions [3].

Developments in molecular epidemiology have allowed rapid identification and tracking specific *Mycobacterium tuberculosis* (MTB) strains spreading through the population. Insertion sequence (IS) ‘6110’ restricted fragment length polymorphism (RFLP), which is the traditional DNA fingerprinting method, is applied to genotype MTB strains and has been utilized in TB outbreak investigation and long-term surveillance for over a decade. However, its wide application is limited because of labor-intensive procedures, demand on a technical level and insufficient discrimination among strains with low IS‘6110’ copy numbers (less than six) [4]. Therefore, several PCR-based methods, such as spoligotyping and MIRU-VNTR, have been well developed and widely used for analyzing genetic diversity and population structure of MTB strains due to the ease of result digitalization, inter-laboratory comparison and data analysis. Spoligotyping, a secondary typing method for MTB strains, is considered the gold standard for identifying Beijing family MTB strains. However, its discriminatory power is limited [5]. Mycobacterial interspersed repetitive units-variable number of tandem repeats (MIRU-VNTR) is based on PCR amplification of the interspersed repetitive units to determine the size and repeated number of each locus. It is a flexible approach, as sizing procedure can be done by capillary [6], gel electrophoresis [7] or nondenaturing high-performance liquid chromatography [8]. Easy operation, digital database and high discriminatory power make it an alternative method for RFLP [9]. Moreover, the combined application of MIRU-VNTR and spoligotyping is increasingly common in MTB molecular epidemiology research [10, 11].

The Hebei province surrounds Beijing and Tianjin, and its strategic position is significantly important due to the development of economic integration of the Beijing-Tianjin-Hebei integration region. The extensive and convenient traffic network makes Hebei a key transportation hub connecting Beijing with the entire country. It covered an area of 188,500 square kilometers with a population of 73.8 million in 2014. Based on the 2000 National TB Epidemiology Survey in China, there were approximately 200,000 pulmonary TB patients in Hebei [12]. The TB epidemic in Hebei leaves still little optimism, and the population mobility and the spread of HIV makes the situation even worse. Moreover, little information about the genetic diversity of *Mycobacterium tuberculosis* in this district is available to date. Therefore, this study aimed to investigate the molecular epidemiological characteristics and drug resistance status of clinical isolated TB strains in Hebei and explore the correlation between genotypes and drug resistance phenotypes.

## Methods

### Description of strains

The study contained 1017 MTB isolates, randomly collected from 1017 sputum samples of 1017 patients, who were clinically confirmed with pulmonary TB in hospitals of infectious diseases in Hebei during 2014. All the isolates were stored at −80 °C and recovered on the Lowenstein-Jensen (L-J) medium at 37 °C for 4 weeks when used. MTB H37Rv, kindly provided by China Center for Disease Control and Prevention, was used as the reference strain.

### DNA extraction

Chromosomal DNA was extracted from mycobacterial colonies grown on L-J medium using Mericon™ DNA Bacteria Kit (Qiagen, USA) according to the manufacturer’s instructions. One loop of MTB colonies was resuspended in 200 μl fast lysis buffer by brief and vigorous vortex, and then incubated at 100 °C for 10 min. After centrifugation at 12,000 rpm for 5 min, the supernatant containing DNA was collected and stored at −20 °C for further use.

### Drug susceptibility testing

The first line anti-TB drug susceptibility testing (DST) was performed using the L-J proportion method, recommended by WHO [13], at the Clinical Laboratory of the Fifth Hospital of Shijiazhuang, China. The critical concentration were 0.2 μg/ml for isoniazid (INH), 40 μg/ml for rifampicin (RIF), 4 μg/ml for streptomycin (STR) and 2 μg/ml for ethambutol (EMB). H37Rv strain was used as a quality control and was tested each batch of DST. The results were read 28 days after inoculation of the strains. When the growth rate was more than 1 % compared to the control, the strain was considered resistant to the specific drug. Strains resistant to at least RIF and INH were defined as MDR-TB.

### Spoligotyping

Spoligotyping was performed using the standard protocol described by Kamerbeek et al. [14]. First, the direct repeat (DR) region was amplified with primers of DRa (biotin-labeled) and DRb. And then, the PCR products were hybridized to a membrane with a set of 43 different oligonucleotide probes covalently bound to the surface. The results in binary format were compared against the international spoligotype database SpolDB4.0 (http://www.pasteur-guadeloupe.fr:8081/SITVIT_ONLINE/) to obtain the spoligotyping pattern [15]. In this database, Spoligotype International Type (SIT) designates an identical pattern shared by two or more patient isolates, whereas orphan designates patterns reported for a single isolate that does not correspond to any of the strains recorded in the database repository [16].

### MIRU-VNTR typing

The 15-locus MIRU-VNTR typing method, including ETRA, ETRC, ETRD, ETRE, MIRU10, MIRU16, MIRU26, MIRU40, Mtub21, Mtub30, Mtub39, Mtub04, QUB11b, QUB26 and QUB4156, was carried out to determine the composition of each isolate [17]. First, the PCR amplification of each locus was carried out individually using primers in Additional file 1. Each PCR reaction contained 10 μl Taq MasterMix (CWBIO, China), 1 μl (10 μM) forward primer, 1 μl (10 μM) reverse primer, 40-60 ng of DNA template and double-distilled H_2_O to bring the final volume to 20 μl. PCR was performed as follows: initial denaturation at 94 °C for 5 min, and then 35 cycles of denaturation at 94 °C for 30 s, annealing at 62 °C for 30 s and extension at 72 °C for 45 s, with a final extension at 72 °C for 10 min. Then, the PCR products were electrophoresed on 1.5 % agarose gel and sized with 100 bp DNA ladder (CWBIO, China). H37Rv was used as positive control and double-distilled H_2_O was used as negative control. The copy number of repeats was calculated and entered into an Excel spreadsheet. In addition, the Hunter-Gaston discriminatory Index (HGDI) was used to evaluate the discriminatory power of each locus.

### Data management

Spoligotyping data in binary format and MIRU-VNTR data in decimal format were analyzed using BioNumerics 5.0 software (Applied-Maths, Sint-Martens-Latem, Belgium). Dendrogram and minimum spanning tree were generated for clustering analysis. The discriminatory power and allelic diversity of each locus were determined by Hunter-Gaston discriminatory index (HGDI) value:

$$ \mathrm{HGDI}=1-\left[\frac{1}{\mathrm{N}\left(\mathrm{N}-1\right)}{\displaystyle \sum_{\mathrm{j}=1}^{\mathrm{s}}{\mathrm{n}}_{\mathrm{j}}\left({\mathrm{n}}_{\mathrm{j}}-1\right)}\right] $$

where N is the total number of strains in the typing method, s is the total number of different patterns discriminated by that method, and n_j_ is the number of strains belonging to the j^th^ pattern. The HGDI value was calculated using the discriminatory power calculator available at http://www.hpa-bioinfotools.org.uk/cgi-bin/DICI/DICI.pl [18]. The clustering rate was defined as (n_c_−c)/N, where n_c_ is the total number of clustered strains, c is the number of clusters and N is the total number of strains.

### Statistical analysis

The statistical analysis was done by SPSS 16.0 software. Chi-square test or Fisher’s exact probability test was used to compare the proportions of different groups. Two-sided *p*-value of less than 0.05 was considered statistically significant.

## Results

### Study population

**Fig. 1:**
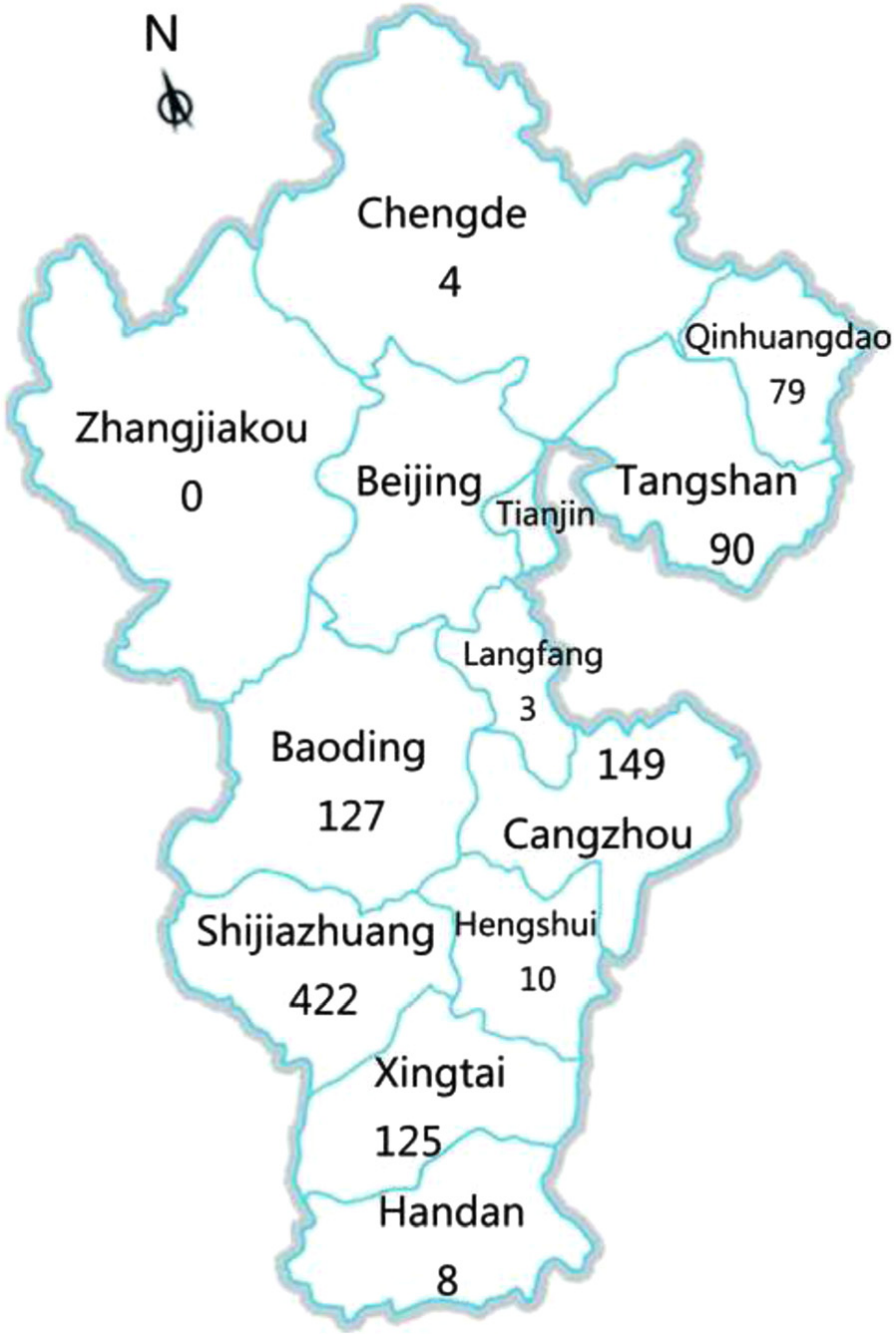
Map of Hebei province showing the distribution of 1017 *M. tuberculosis* isolates enrolled in this study. The numbers represent the population of isolates in particular regions'

A total of 1017 MTB strains collected from various regions of the Hebei province during 2014 were enrolled in this study, including 422 strains from Shijiazhuang, 149 from Cangzhou, 127 from Baoding, 125 from Xingtai, 90 from Tangshan, 79 from Qinhuangdao, 10 from Hengshui, 8 from Handan, 4 from Chengde, 3 from Langfang and none from Zhangjiakou (Fig. 1). The enrolled patients included 705 males (69.3 %) and 312 females (30.7 %), with a median age of 38 (range from 11 to 89) years. The anti-TB drug treatment history was available for 974 patients, including 753 new cases and 221 retreatment patients. In addition, 493 of the new cases and 181 of the retreatment patients were males.

### Spoligotyping

Among the 1017 strains genotyped, 72 spoligotypes were identified with the HGDI of 0.279. Of these, 57 spoligotypes were previously represented in the SpolDB4.0 database, while the other 15 were not found in the database and were marked as New genotypes. Out of the 57 unique spoligotypes, 7 orphan spoligotypes were identified, which referred to an isolate having a spoligotype that was present only once in the database. An overview of New and orphan strains can be found in Additional file 2.

The family assignment revealed that 920 strains (90.5 %) belonged to the Beijing family, 97 (9.5 %) were from the non-Beijing family. The non-Beijing family included 39 strains from the T1 family, 15 from the T2 family, 5 from the T3 family, 1 from the T4 family, 1 from the T5 family, 1 from the LAM1 family, 3 from the LAM9 family, 7 from the MANU2 family, 7 from the H3 family, 1 from the U family, 3 from the AMBIGOUS: T3 T2 family and 14 from undefined family (Table 1). The clustering analysis grouped 972 strains (95.6 %) into 27 clusters, and the remaining 45 (4.4 %) were unique genotypes. The largest cluster belonged to the typical Beijing family, containing 863 strains (84.9 %) with the SIT number of 1, characterized by the absence of the first 34 spacers and the presence of spacers 35 to 43. The other 55 strains (5.4 %) were defined as the atypical Beijing family for missing one or more dots in spacers 35 to 43. Among 97 strains of the non-Beijing family, 20 with the SIT number of 53 comprised the biggest cluster of the non-Beijing family. The dendrogram also suggested that 2 of the New genotypes were clustered into the atypical Beijing family, and 13 were grouped into the non-Beijing family (Additional file 3).

**Table 1:** Spoligotypes of *Mycobacterium tuberculosis* strains in this study (*n* = 1017)

No.	Spoligotype	SIT^a^	Family^b^	N(%)^c^
1	□□□□□□□□□□□□□□□□□□□□□□□□□□□□□□□□□□■■■■■■■■■	1	Beijing	863(84.9)
2	□□□□□□□□□□□□□□□□□□□□□□□□□□□□□□□□□□■■■■■□■■■	190	Beijing	18(1.8)
3	□□□□□□□□□□□□□□□□□□□□□□□□□□□□□□□□□□■■■■□■■■■	255	Beijing	2(0.2)
4	□□□□□□□□□□□□□□□□□□□□□□□□□□□□□□□□□□■■□□■■■■■	260	Beijing	4(0.4)
5	□□□□□□□□□□□□□□□□□□□□□□□□□□□□□□□□□□■■□■■■■■■	265	Beijing	3(0.3)
6	□□□□□□□□□□□□□□□□□□□□□□□□□□□□□□□□□□□□■■■■■■■	269	Beijing	6(0.6)
7	□□□□□□□□□□□□□□□□□□□□□□□□□□□□□□□□□□□□■■■□■■■	406	Beijing	1(0.1)
8	□□□□□□□□□□□□□□□□□□□□□□□□□□□□□□□□□□■■■■■□□■■	541	Beijing	2(0.2)
9	□□□□□□□□□□□□□□□□□□□□□□□□□□□□□□□□□□■□■■■■■■■	621	Beijing	4(0.4)
10	□□□□□□□□□□□□□□□□□□□□□□□□□□□□□□□□□□■■■□■■■■■	632	Beijing	2(0.2)
11	□□□□□□□□□□□□□□□□□□□□□□□□□□□□□□□□□□■■■■■■□■■	941	Beijing	2(0.2)
12	□□□□□□□□□□□□□□□□□□□□□□□□□□□□□□□□□□■■■■■□□□□	1311	Beijing	2(0.2)
13	□□□□□□□□□□□□□□□□□□□□□□□□□□□□□□□□□□■■■□□■■■■	1364	Beijing	1(0.1)
14	□□□□□□□□□□□□□□□□□□□□□□□□□□□□□□□□□□■■■■■■■□■	1674	Beijing	1(0.1)
15	□□□□□□□□□□□□□□□□□□□□□□□□□□□□□□□□□□■■□□□□□□□	2101	Beijing	3(0.3)
16	□□□□□□□□□□□□□□□□□□□□□□□□□□□□□□□□□□■■■■■■■■□	2610	Beijing	2(0.2)
17	□□□□□□□□□□□□□□□□□□□□□□□□□□□□□□□□□□■■■□□□■■■	Orphan	Beijing	1(0.1)
18	□□□□□□□□□□□□□□□□□□□□□□□□□□□□□□□□□□■■■□■□■■■	Orphan	Beijing	1(0.1)
19	■■■■■■■■■■■■■■■■■■■■■■■■■■■■■■■■□□□□■■■■■■■	53	T1	20(2.0)
20	■■■■■■■■■■■■■■■■■■■■■■■■■■■■■■■■□□□□□■■■■■■	240	T1	1(0.1)
21	■■■■■■■■■■■■■■■■■■■■■■■■■■■■■■■■□□□□□□□□□■■	956	T1	2(0.2)
22	■□■■■■■■■■■■■■■■■■■■■■■■■■■■■■■■□□□□■■■■■■■	334	T1	2(0.2)
23	■■■■■■■■■■■■■■■■■■■■■■■■■■■■■■■■□□□□□□□□□□□	1793	T1	1(0.1)
24	■■■■■■■■■■■□■■■■■■■■■■■■■■■■■■■■□□□□■■■■■■■	498	T1	1(0.1)
25	■■■■■■■■■■■■□□■■■■■■■■■■■■■■■■■■□□□□■■■■■■■	131	T1	1(0.1)
26	■■■■■■■■■■■■■■■■■■■□□□□□□■■■■■■■□□□□■■■■■■■	1688	T1	1(0.1)
27	■■■■■■■■■■■■■■■■■■■■□■■■■■■■■■■■□□□□■■■■■■■	291	T1	1(0.1)
28	■■■■■■■■■■■■■■■■■■■■■■■■■■□■■■■■□□□□■■■■■■■	1626	T1	1(0.1)
29	■■■■■■■■■■■■■■■■■■■■■■■■■■■■□■■■□□□□■■■■■■■	462	T1	1(0.1)
30	■■■■■■■■■■■■■■■■■■■■■■■■■■■■■□■■□□□□□□□■■■■	Orphan	T1	2(0.2)
31	■■■■■■■■■■■■■■■■■■■■■■■■■■■■■■■■□□□□■□■■■■■	520	T1	1(0.1)
32	■■■■■■■■■■■■■■■■■■■■■■■■■■■■■■■■□□□□■■■■■■□	522	T1	3(0.3)
33	■■■■■■□□□□□□□□□□□□■■■■■■■■■■■■■■□□□□■■■■■■■	280	T1-RUS2	1(0.1)
34	■■■■■■■■■■■■■■■■■■■■■■■■■■■■■■■■□□□□■■■□■■■	52	T2	9(0.9)
35	■■■□■■■■■■■■■■■■■■■■■■■■■■■■■■■■□□□□■■■□■■■	848	T2	1(0.1)
36	□□■■■■■■■■■■■■■■■■■■■■■■■■■■■■■■□□□□■■■□■■■	1613	T2	1(0.1)
37	■■■■■■■■■■■■■■■■■■■■■■■■■■■■■■■■□□□□□□■□■■■	Orphan	T2	1(0.1)
38	■■■■■■■■■■■■■■■■■■■■■■■□■■■■■■■■□□□□■■■□■■■	Orphan	T2	1(0.1)
39	■■■■■■■■■■■■■■■■■■■■■■■■□□□□□□■■□□□□■■■□■■■	233	T2	2(0.2)
40	■■■■■■■■■■■■□■■■■■■■■■■■■■■■■■■■□□□□■■■■■■■	37	T3	2(0.2)
41	■□■■■■■■■■■■□■■■■■■■■■■■■■■■■■■■□□□□■■■■■■■	Orphan	T3	3(0.3)
42	■■■■■■■■■■■■■■■■■■□■■■■■■■■■■■■■□□□□■■■■■■■	40	T4	1(0.1)
43	■■■■■■■■■■■■■■■■■■■■■■□■■■■■■■■■□□□□■■■■■■■	44	T5	1(0.1)
44	■□□□□□■■■■■■■■■■■■■■□□□□■■■■■■■■□□□□■■■■■■■	961	LAM1	1(0.1)
45	■■■■■■■■■■■■■■■■■■■■□□□□■■■■■■■■□□□□■■■■■■■	42	LAM9	1(0.1)
46	■■■■■■■■■■■■■□□□□□□□□□□□■■■■■■■■□□□□■■■■■■■	803	LAM9	1(0.1)
47	■■■■■■■■■■■■■□□□□■□□□□□□■■■■■■■■□□□□■■■■■■■	2191	LAM9	1(0.1)
48	■■■■■■■■■■■□□□□□□□□□□□□□□□□□□□□□□□□□□□□□□□□	2379		1(0.1)
49	■■■■■■■■■■■■■■■■■■■■■■■■■■■■■■■■□□■■■■■■■■■	54	MANU2	4(0.4)
50	■■■■■■■■■■■■■■■■■■■■□■■□■■■■■■■■□□■■■■■■■■■	1523	MANU2	1(0.1)
51	■■■■■■■■■■■■■■■■■■■■□■■■■■■■■■■■□□■■■■■■■■■	1192	MANU2	2(0.2)
52	■■■■■■■■■■■■■■■■■■■■■■■■■■■■■■□■□□□□■■■■■■■	50	H3	3(0.3)
53	■■■■■■■■■■■■■■■■■■■■□■■■■■■■■■□■□□□□■■■■■■■	268	H3	2(0.2)
54	■■■■■■■■■■■■■■■■■■■■■■■□□□□□□□□■□□□□■■■■■■■	791	H3	1(0.1)
55	■■■■■■■■■■■■■■■■■■■■■■■■■■□□□□□■□□□□■■■■■■■	1908	H3	1(0.1)
56	■■■■■■■■■■■■■■■■■■■■■■■■□□□□□□□□□□□□■■■■■■■	602	U	1(0.1)
57	■□■■■■■■■■■■□■■■■■■■■■■■■■■■■■■■□□□□■■■□■■■	Orphan	AMBIGOUS:T3T2	3(0.3)
58	■■■■■■■□■■■■■■■■□■■■□■■■■■■■■■■■□□■■■■■■■■■	-	New	1(0.1)
59	■■■■■■■■■■■■■■■■■■■■■■■■■□■■■■■■□□□□□□■■■■■	-	New	1(0.1)
60	■□□■■■□□■■■■■■■■■■■■■■■□□■■■□□□□□□■■□□□■□■■	-	New	1(0.1)
61	■■■■□■■■■■■■■■■■■■■■■■■■■■■■□□□□□□□□□□□□□□□	-	New	1(0.1)
62	■■■■■■■■■■■■□□■■■■■■■■■■■■■□■□■■□□■■■■■■■■■	-	New	1(0.1)
63	□□□□■■■■■■■■■■■■■■■■■■■■■■■■■■■□□□□□■■■□■■■	-	New	1(0.1)
64	■■■■■■■■■■■■□■■■■■■■■■■■■■■■■□■■□□□□■■□■■■■	-	New	1(0.1)
65	■□■■■■■■■■■■□■■■□■■■■■■■■■■■■■■■□□□□■■■■■■■	-	New	1(0.1)
66	■□□□□□□■■■■□□■■■■■■■■■■■■■■■■■■■□□□□■■■■■■■	-	New	1(0.1)
67	■□■■■■■■■■■■□□□□■■■■■■■■■■■■■■■■□□□□■■□□■■■	-	New	1(0.1)
68	■□■■■■■■■■■■■■■■■■□■■□□□■■■■■■■■■■■■■■■□■■■	-	New	1(0.1)
69	■■■■■■□□□■■■■■■■■■■■■■□□□□□□□□□■□□□□■■■■■■■	-	New	1(0.1)
70	■■■■■■■■■■■■■■■■■■■■■■■■■■□□■■■□□□□□□□□■■■□	-	New	1(0.1)
71	□□□□□□□□□□□□□□□□□□□□□□□□□□□□□□□□□□■■■■■□■□■	-	New	1(0.1)
72	□□□□□□□□□□□□□□□□□□□□□□□□□□□□□□□□□□■■■□□□□□□	-	New	1(0.1)

^a^ *SIT* spoligotype international type

^b^Spoligotype families as assigned in SpolDB4.0

^c^The number of the strains with a common SIT

**Fig. 2:**
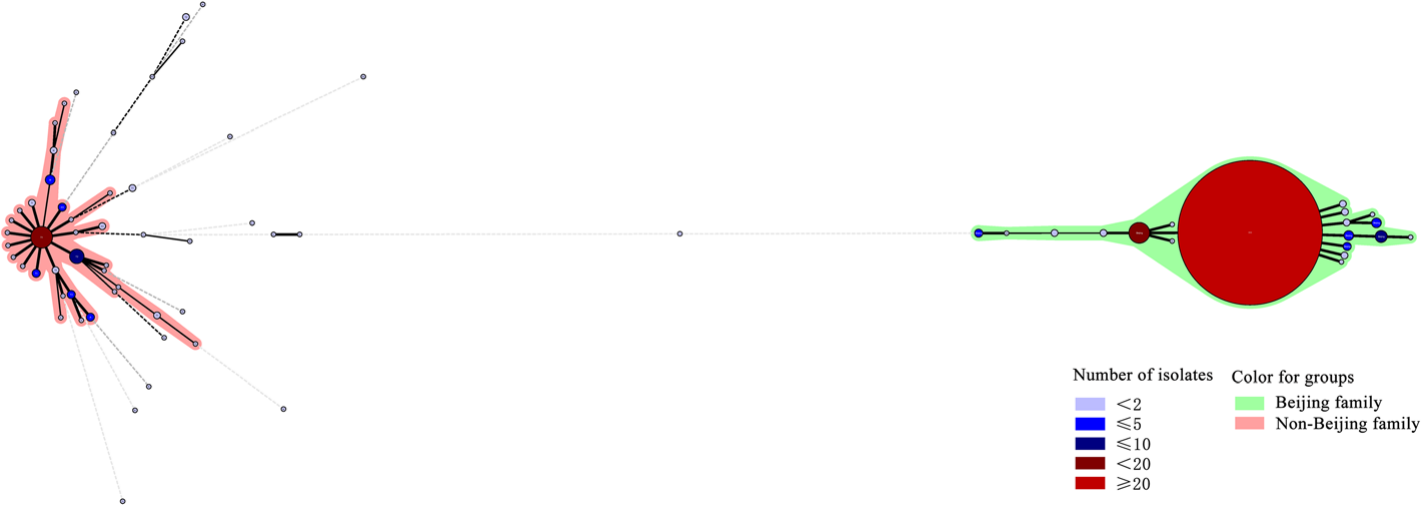
Minimum spanning tree showing the clustering analysis of 1017 *M. tuberculosis* strains from Hebei by spoligotyping

The minimum spanning tree was mapped to describe the genetic links between the clusters using BioNumerics 5.0 software. Each node represented a particular genotype, and the size of each node was determined by the number of strains within that genotype [19]. As shown in Fig. 2, the Beijing family and the non-Beijing family were classified into two large groups. The biggest cluster containing 863 strains (SIT1) belonged to the typical Beijing family and was surrounded by small clusters of the atypical Beijing family. The two larger clusters on the left were the T1 and T2 family with the other non-Beijing family and 13 New genotypes around them. Particularly, 2 New genotyped strains were genetically close to the Beijing family and the other 13 New genotypes were similar to the non-Beijing family.

### MIRU-VNTR typing

**Fig. 3:**
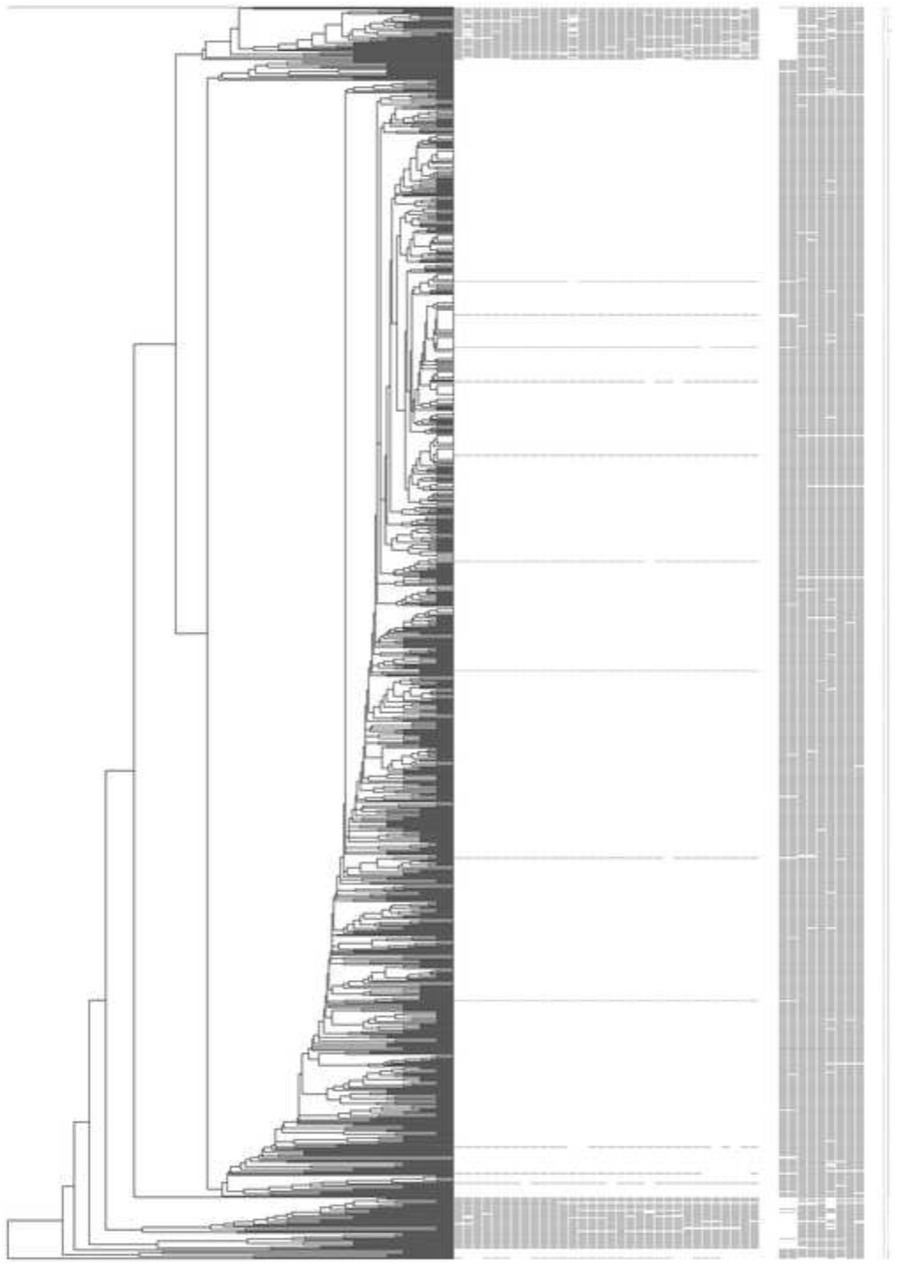
Genotyping of 1017 *M. tuberculosis* strains with MIRU-VNTR and spoligotyping. From *left* to *right*: UPGMA dendrogram generated by MIRU-VNTR, spoligotyping patterns, strain numbers, genetic lineage based on SpolDB4.0

The 15-locus MIRU-VNTR clustering analysis divided 255 strains (25.1 %) into 84 clusters (2-17 strains per cluster) and the remaining 762 (74.9 %) were individual patterns. The clustering rate was 16.8 %. The clade assignment showed that all the 1017 MTB strains were clustered into 16 gene groups (Fig. 3). Groups I, II and III shared similar MIRU-VNTR profiles, demonstrated high homology with a close evolutionary distance and belonged to the non-Beijing family according to spoligotyping. Group IV contained 17 individual genotypes, and all of them are members of the Beijing family strains. Group IV was closely related to Group V, the largest clade, including 894 Beijing family isolates and 12 non-Beijing family strains. There were 82 clusters in Group V and the biggest cluster, which was composed of 16 Beijing family genotypes and 1 T1 strain, shared the same MIRU-VNTR fingerprint, 643333564475493. One Beijing family strain was classified into Group VI, as four out of 15 loci were homologous with either Group V or VII. Groups VII, VIII and IX contained 29 non-Beijing family strains with similar MIRU-VNTR fingerprints and close evolutionary distance. Among them, seven strains belonged to T1, 14 to T2, 2 to MANU2, 2 to LAM9, 3 to New genotypes and 1 to an untitled family with a SIT number of 2379. As the dendrogram showed that the SIT2379 strain was genetically close to T2 family and the 3 New genotypes shared high homology with T1 and T2 family. The spoligotypes of Group X and XI belonged to T1 family except 1 MANU2 family isolate. Group XII ~ XV included seven individual Beijing family strains, characterized by 2 ~ 4 loci absent and 6 ~ 9 loci identical with strains in Group V. The independent New spoligotyped strain in Group XVI demonstrated a particular MIRU-VNTR profile with little similarities to strains in other groups.

**Fig. 4:**
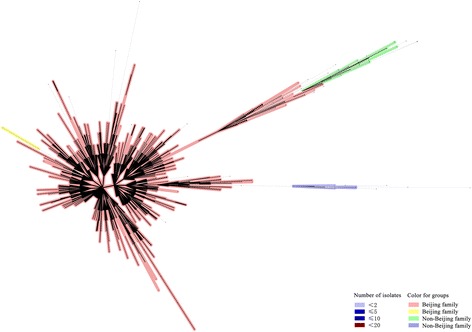
Minimum spanning tree showing the clustering analysis of 1017 *M. tuberculosis* strains from Hebei by MIRU-VNTR

The minimum spanning tree showed that the polymorphism of MTB isolates enrolled was intensively high (Fig. 4). The largest cluster belonged to the Beijing genotype (light red and yellow) and was separated from the non-Beijing family (green and blue). MIRU-VNTR can separate the Beijing family into different small branches, indicating that it possessed a higher discriminatory power than spoligotyping.

The 15 MIRU-VNTR loci exhibited different HGDI scores, ranging from 0.052 for ETRC to 0.722 for QUB11b. Based on the HGDI values, the loci were further classified into highly (>0.6), moderately (0.3-0.6) and poorly (<0.3) discriminative loci [20, 21]. For all the strains, the HGDI of 2 loci (QUB11b and QUB26) were higher than 0.6, 7 loci (QUB4156, ETRE, Mtub21, Mtub04, MIRU26, MIRU10 and ETRA) showed moderately discriminatory power and the other 6 loci (MIRU40, MIRU16, Mtub39, Mtub30, ETRD and ETRC) were found to be less discriminative (Table 2). The discriminatory power (HGDI) of the total loci set reached 0.999.

**Table 2:** HGDI scores of the MIRU-VNTR loci for all the strains

Locus	HGDI	95 % CI	No. of alleles
QUB11b	0.722	0.714–0.730	12
QUB26	0.651	0.638–0.665	16
QUB4156	0.594	0.583–0.606	8
ETRE	0.592	0.579–0.605	10
Mtub21	0.555	0.539–0.570	11
Mtub04	0.554	0.539–0.568	9
MIRU26	0.547	0.531–0.563	12
MIRU10	0.407	0.391–0.424	8
ETRA	0.372	0.357–0.387	8
MIRU40	0.289	0.272–0.306	9
MIRU16	0.222	0.206–0.238	6
Mtub39	0.207	0.191–0.222	10
Mtub30	0.190	0.175–0.204	7
ETRD	0.171	0.156–0.186	10
ETRC	0.052	0.043–0.061	6

### Drug resistance

The drug susceptibility testing showed that 670 isolates (65.9 %) were sensitive to the four first-line anti-TB drugs tested and 347 isolates (34.1 %) were resistant to at least one of these drugs, including the 134 (13.2 %) MDR strains. The drug resistance rates of STR, INH, RIF, EMB were 24.9 % , 20.6 %, 17.9 % and 8.4 %, respectively (Table 4). The total drug resistance rate of the retreatment patients were significantly higher than that of the initial treatments, and the same results were seen in STR, INH, RIF and EMB mono-resistance rate and MDR rate between the two groups (Table 3). However, the Beijing and non-Beijing family had no statistical associations in any drug resistance rates (Table 4).

**Table 3:** Drug susceptibility phenotypes of new cases and retreatments

DST^a^	*N* (%)	New cases	Retreatments	OR (95 % CI)^b^	*p-*value
Total	974	753 (77.3 %)	221 (22.7 %)		
Pan sensitive	642 (65.9 %)	553 (86.1 %)	89 (13.9 %)	1 (Ref.)	
Any resistance^c^	332 (34.1 %)	200 (60.2 %)	132 (39.8 %)	4.101 (2.996–5.613)	<0.0001
Any S resistance	240 (24.6 %)	137 (57.1 %)	103 (42.9 %)	4.671 (3.325–6.562)	<0.0001
Any H resistance	201 (20.6 %)	96 (47.8 %)	105 (52.2 %)	6.796 (4.760–9.703)	<0.0001
Any R resistance	173 (17.8 %)	77 (44.5 %)	96 (55.5 %)	7.747 (5.328–11.26)	<0.0001
Any E resistance	84 (8.6 %)	41 (48.8 %)	43 (51.2 %)	6.517 (4.020–10.56)	<0.0001
MDR^d^	129 (13.2 %)	46 (35.7 %)	83 (64.3 %)	11.21 (7.335–17.14)	<0.0001

^a^ *DST* drug susceptibility testing

^b^ *OR* odds ratio, *CI* confidence interval

^c^ *S* streptomycin, *H* isoniazid, *R* rifampicin, *E* ethambutol

^d^ *MDR* multi-drug resistance

**Table 4:** Drug susceptibility phenotypes of the Beijing and non-Beijing family strains

DST^a^	*N* (%)	Beijing, *n* (%)	Non-Beijing, *n* (%)	OR (95 % CI)^b^	*p-*value
Total	1017	920 (90.5 %)	97 (9.5 %)		
Pan sensitive	670 (65.9 %)	605 (90.3 %)	65(9.7 %)	1 (Ref.)	
Any resistance^c^	347 (34.1 %)	315 (90.8 %)	32 (9.2 %)	0.946(0.606-1.475)	0.910
Any S resistance	253 (24.9 %)	229 (90.5 %)	24 (9.5 %)	0.976(0.596-1.596)	1.000
Any H resistance	210 (20.6 %)	191 (91.0 %)	19 (9.0 %)	0.926(0.541-1.583)	0.893
Any R resistance	182 (17.9 %)	169 (92.9 %)	13 (7.1 %)	0.716(0.385-1.330)	0.314
Any E resistance	85 (8.4 %)	75 (88.2 %)	10 (11.8 %)	1.241(0.611-2.519)	0.563
MDR^d^	134 (13.2 %)	123 (91.8 %)	11 (8.2 %)	0.832(0.427-1.623)	0.746

^a^ *DST* drug susceptibility testing

^b^ *OR* odds ratio, *CI* confidence interval

^c^ *S* streptomycin, *H* isoniazid, *R* rifampicin, *E* ethambutol

^d^ *MDR* multi-drug resistance

### Comparison of clinical factors and clustering characteristics between the Beijing and non-Beijing family

Clinical factors, including age, gender and treatment history were analyzed between the Beijing and non-Beijing family, but no statistical difference was found (Table 5). In particular, compared to the initial treatment group, men accounted for a significantly larger proportion of the retreatment patients than women [*p* < 0.0001; OR (95 %): 2.386 (1.642–3.469)]. The Beijing family strains exhibited a significantly higher clustering rate than the non-Beijing family strains by both spoligotyping and MIRU-VNTR typing methods (Table 6).

**Table 5:** Clinical characteristics of the Beijing and non-Beijing family strains

Characteristics	Total	Beijing family	Non-Beijing family	OR (95 % CI)	*p*-value
Age, years					
< 30	424	389	35	-	0.434
30–60	404	362	42		
> 60	189	168	21		
Gender					
Male	705	632	73	1	0.204
Female	312	288	24	0.722 (0.446–1.168)	
Treatment history					
New case	753	685	68	1	0.433
Retreatment	221	197	24	1.277 (0.751–2.007)

**Table 6:** Comparison of clustered and individual strains between the Beijing and non-Beijing family

Genotyping	Genotype	Beijing family	Non-Beijing family	*p*-value
Spoligotyping	Individual strains, *n*	7	38	
	Clustered strains, *n*	913	59	<0.0001
	Clusters, *n*	13	14	
	Clustering rate, %	97.8	46.4	
MIRU-VNTR	Individual strains, *n*	669	93	
	Clustered strains, *n*	251	4	<0.0001
	Clusters, *n*	82	2	
	Clustering rate, %	18.4	2.1	

## Discussion

This is the first study on the genetic diversity of *Mycobacterium tuberculosis* strains in Hebei by two widely used genotyping methods, spoligotyping and MIRU-VNTR typing. Our study found that the Beijing family is the predominant lineage of MTB strains in Hebei, lower than that of Beijing (92.6 %) and Tianjin (91.7 %) [22, 23]. In addition, non-Beijing family, including T1, T2, T3, T4, T5, LAM1, LAM9, H3, U, MANU2, and AMBIGOUS: T3 T2, demonstrated the genotypic polymorphisms of MTB strains in Hebei. Based on the SpolDB4.0 database, one strain with the SIT number of 2379, which was first isolated from Belgium in 2004, belonged to an untitled family. This is the first report that SIT2379 strain has been isolated from China.

The Beijing family MTB strains have attracted worldwide attention for their wide geographical distribution and global emergence. This lineage has been reported to be the most prominent clade in East Asia, China, Korea and Japan, and also enjoyed popularity in Europe, Africa, and North America [24–26]. The apparent global success of the Beijing lineage suggested that it might have selective advantages (higher virulence or transmissibility) over other MTB genotypes. Additionally, it was hypothesized that the Beijing family strains can escape from the protection of BCG vaccine and the widespread of Beijing genotype may result in the selective force of widely BCG vaccination [27]. In our study, Beijing strains were significantly associated with genotypic clustering, reflecting recent transmission of this lineage in Hebei and consistent with the former viewpoint.

Evidence has shown that the mutations in DNA repair genes provided a selective advantage for Beijing genotype strains to acquire resistance to anti-TB drugs [28]. However, the association between the Beijing lineage and drug resistance varied. In New York, Cuba, Estonia, and Vietnam, the Beijing strains were strongly associated with drug resistance, but elsewhere the association was weak or absent [26]. Consistent with the latter, our finding considered that the Beijing family genotypes were no more likely to acquire drug resistance than the non-Beijing genotypes. The results were probably biased by the different proportion of Beijing subgroup in different areas and the different ratios of ancient/modern Beijing strains within different study population [29]. More detailed studies on the drug resistance mechanisms of particular genotype strains are of the utmost importance. In addition, the statistical analysis demonstrated that the Beijing and non-Beijing family exhibited no difference in distribution of gender, age, and treatment history, indicating that other factors may contribute to the widespread of the Beijing family strains.

In the present study, the drug resistance rate of the retreatment patients was significantly higher than that of new cases, indicating that improper use of TB antibiotics, including incorrect treatment regimens and poor treatment compliance of TB patients, may result in drug resistance. Advocating the rational use of anti-TB drugs in clinical practice contributes to reduce the emergence of drug resistant MTB strains.

A 70 % excess of male over female TB cases is reported each year in the world [30]. It was reported that the global male/female sex ratio was 1.96, with the highest being 4.93 in Russia and range of 0.8–1.13 observed in Central America, Caribbean, Eastern Africa and Northern Europe [31]. The male/female sex ratio in Hebei reached 2.26 (705/312), which is higher than the average level. A strong opinion claimed the gender bias in TB infection, which means that the prevalence of TB was higher in men than in women [32]. In comparison to new cases, we found that the proportion of males in the retreatment panel was significantly larger than females. This suggested that men were at a higher risk of TB recurrence. However, the reason for this difference and the role of Y chromosome on the susceptibility of MTB are still unclear.

Owing to the fairly low discriminatory power of spoligotyping, 15 locus MIRU-VNTR was conducted for further analysis. Data showed that the allelic diversity of 15 loci varied significantly, from 0.052 for ETRC to 0.722 for QUB11b, similar to the report from Inner Mongolia [21]. The Beijing family strains with identical MIRU-VNTR profiles were grouped into small branches, but the non-Beijing family exhibited a high genetic diversity of MIRU-VNTR profiles, partly due to the small population size. We hypothesized that the transmission ability of the Beijing family was stronger than the non-Beijing family in Hebei, but more epidemiology studies are needed to confirm it.

Our study had some limitations. First, the number of strains from Zhangjiakou, Chengde, Langfang, Hengshui and Handan was quite small, because of no particular TB laboratory in the local hospitals due to the poor medical facilities and lack of technicians. Second, more clinical factors needed to be collected and analyzed to reveal the characteristics of different genotypes better. Finally, mutations of drug resistance gene should be detected to understand the genetic diversity of strains in Hebei fully.

## Conclusions

In summary, the MTB strains demonstrate high genetic diversity and the predominant lineage is the Beijing family in Hebei. The Beijing family is associated with genetic clustering, but no correlation is observed with gender, age, treatment history and drug resistance profiles. Spoligotyping in combination with 15-locus MIRU-VNTR is a useful tool to study the molecular epidemiology of MTB strains in this area.

### Ethics statement

This study was approved by the Ethics Committee of the Fifth Hospital of Shijiazhuang, Hebei, China. All the patients were given written informed consent form and signed to participate in this study. For patients under 16 years of age, the consent was obtained from their parents or guardians.

### Availability of data and materials

The online version of this article contains supplementary material, which is available to authorized users.

## Additional files

Additional file 1: Primer information of the MIRU-VNTR loci in this study. (DOCX 15 kb) Additional file 2: Detailed information about New and orphan spoligotype patterns isolated from 1017 MTB strains in Hebei. (XLSX 15 kb) Additional file 3: Genotyping of 1017 *M. tuberculosis* strains with spoligotyping. From left to right: UPGMA dendrogram generated by spoligotyping, spoligotyping patterns, strain numbers, genetic lineage based on SpolDB4.0. (PDF 163 kb)

## Abbreviations

DR: direct repeat
DST: drug susceptibility testing
EMB: ethambutol
HGDI: Hunter-Gaston discriminatory Index
INH: isoniazid
IS: insertion sequence
L-J: Lowenstein-Jensen
MDR-TB: multi-drug resistance TB
MIRU-VNTR: mycobacterial interspersed repetitive units-variable number of tandem repeats
MTB: *Mycobacterium tuberculosis*
OR: odds ratio
RFLP: restricted fragment length polymorphism
RIF: rifampicin
SIT: spoligotype international type
STR: streptomycin
TB: tuberculosis
WHO: World Health Organization
