# Supplementary material for: Characterization of Mycobacterium tuberculosis isolates from Hebei, China: genotypes and drug susceptibility phenotypes
Source: BMC Infect Dis. 2016 Mar 3;16:107. doi: 10.1186/s12879-016-1441-2 (PMC4778344; doi:10.1186/s12879-016-1441-2)
Supplement: Additional file 1: — Primer information of the MIRU-VNTR loci in this study. (DOCX 15 kb) [file 12879_2016_1441_MOESM1_ESM.docx]

**Additional file 1 Primer information of the MIRU-VNTR loci in this study.**

| **Loci** ^a^ | **Primer** | **Primer sequence** | **Repeat unit size（bp）** | **Predicted size（bp）** |
| --- | --- | --- | --- | --- |
| 2165 | ETRA | L) ATTTCGATCGGGATGTTGAT | 75 | 397 |
|  |  | R)TCGGTCCCATCACCTTCTTA |  |  |
| 577 | ETRC | L)GACTTCAATGCGTTGTTGGA | 58 | 346 |
|  |  | R)GTCTTGACCTCCACGAGTGC |  |  |
| 580 | ETRD | L)GCGCGAGAGCCCGAACTGC | 77 | 330 |
|  |  | R)GCGCAGCAGAAACGTCAGC |  |  |
| 3192 | ETRE | L)ACTGATTGGCTTCATACGGCTTTA | 53 | 651 |
|  |  | R)GTGCCGACGTGGTCTTGAT |  |  |
| 960 | MIRU10 | L)GTTCTTGACCAACTGAGTCGTCC | 53 | 643 |
|  |  | R)GCCACCTTGGTGATCAGCTACCT |  |  |
| 1644 | MIRU16 | L)TCGGTGATCGGGTCCAGTCCAAGTA | 53 | 671 |
|  |  | R)CCCGTCGTGCAGCCCTGGTAC |  |  |
| 2996 | MIRU26 | L)CCCGCCTTCGAAACGTCGCT | 51 | 613 |
|  |  | R)TGGACATAGGCGACCAGGCGAATA |  |  |
| 802 | MIRU40 | L)GGGTTGCTGGATGACAACGTGT | 54 | 407 |
|  |  | R)GGGTGATCTCGGCGAAATCAGATA |  |  |
| 1955 | Mtub21 | L)AGATCCCAGTTGTCGTCGTC | 57 | 206 |
|  |  | R)CAACATCGCCTGGTTCTGTA |  |  |
| 2401 | Mtub30 | L)AGTCACCTTTCCTACCACTCGTAAC | 58 | 319 |
|  |  | R)ATTAGTAGGGCACTAGCACCTCAAG |  |  |
| 3690 | Mtub39 | L)AATCACGGTAACTTGGGTTGTTT | 58 | 515 |
|  |  | R)GATGCATGTTCGACCCGTAG |  |  |
| 2163b | QUB11b | L)CCGATGTAGCCCGTGAAGA | 69 | 574 |
|  |  | R)AGGGTCTGATTGGCTACTCA |  |  |
| 424 | Mtub04 | L)CTTGGCCGGCATCAAGCGCATTATT | 51 | 639 |
|  |  | R)GGCAGCAGAGCCCGGGATTCTTC |  |  |
| 4052 | QUB26 | L)GGCCAGGTCCTTCCCGAT | 111 | 708 |
|  |  | R)AACGCTCAGCTGTCGGAT |  |  |
| 4156 | QUB4156 | L)TGACCACGGATTGCTCTAGT | 59 | 703 |
|  |  | R)GCCGGCGTCCATGTT |  |  |

^a^ Available from MIRU-VNTR*plus* website: http://www.miru-vntrplus.org/MIRU/index.faces.
