# Supplementary figures and images for: Characterization of Mycobacterium tuberculosis isolates from Hebei, China: genotypes and drug susceptibility phenotypes
Source: BMC Infect Dis. 2016 Mar 3;16:107. doi: 10.1186/s12879-016-1441-2 (PMC4778344; doi:10.1186/s12879-016-1441-2)

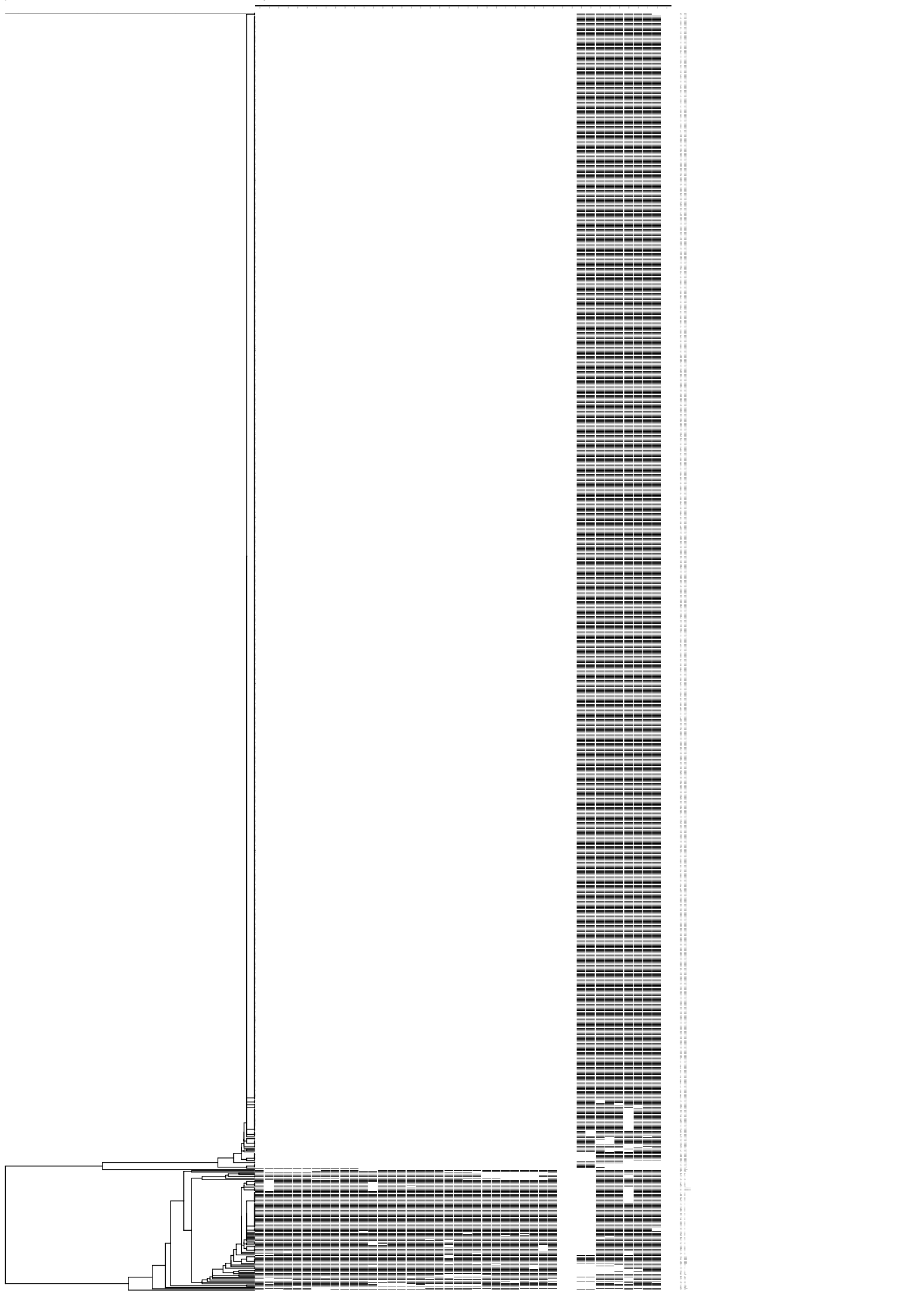

Supplement: Additional file 3: — Genotyping of 1017 M. tuberculosis strains with spoligotyping. From left to right: UPGMA dendrogram generated by spoligotyping, spoligotyping patterns, strain numbers, genetic lineage based on SpolDB4.0. (PDF 163 kb) [file 12879_2016_1441_MOESM3_ESM.pdf]
